# Distinct Transcriptional Responses to 17β-Estradiol in Human Meniscal Fibrochondrocytes from a Female and a Male Donor

**DOI:** 10.3390/ijms27219881

**Published:** 2026-11-04

**Authors:** Youngmin Yu, John Vergis, Hunter M. Eby, Mario Markho, Jonathan Kopacz, Kayla Cartwright, Michael Hershey, Jiayong Liu, Robert E. McCullumsmith

**Affiliations:** 1 Department of Neurosciences and Psychiatry, College of Medicine and Life Sciences, The University of Toledo, Toledo, OH 43614, USA; youngmin.yu@rockets.utoledo.edu (Y.Y.); john.vergis@rockets.utoledo.edu (J.V.); hunter.eby@rockets.utoledo.edu (H.M.E.); mario.markho@rockets.utoledo.edu (M.M.); jonathan.kopacz@rockets.utoledo.edu (J.K.); kayla.cartwright@rockets.utoledo.edu (K.C.); michael.hershey@rockets.utoledo.edu (M.H.); 2 Department of Orthopaedic Surgery, College of Medicine and Life Sciences, The University of Toledo, Toledo, OH 43614, USA; jiayong.liu@utoledo.edu; 3 ProMedica, Toledo, OH 43604, USA

**Keywords:** osteoarthritis, meniscus, meniscal fibrochondrocytes, 17β-estradiol, sex differences, transcriptomics, gene set enrichment analysis, NOTCH1, matrix metalloproteinases

## Abstract

Osteoarthritis (OA) affects women more often than men, and estrogen has been implicated in joint homeostasis, yet its effects on meniscal fibrochondrocytes (MFCs) remain poorly defined. We reanalyzed a public RNA sequencing dataset (Gene Expression Omnibus, GSE199087) of MFCs from two age-matched donors, one female and one male (both 47 years old). Cells from each donor were treated in triplicate with pulsed 17β-estradiol (E2, 240 µM for 1 h every 24 h over 72 h) or vehicle control. Differential expression was assessed with limma-voom, and pathways were identified with Gene Set Enrichment Analysis (GSEA), Enrichr, and iLINCS, retaining only Gene Ontology terms supported by all three. In the female donor’s cells, E2 upregulated DNA replication and cell-cycle pathways and lowered *MMP1*, *MMP2*, *IL12A*, and *MYD88*. In the male donor’s cells, E2 upregulated chromatin remodeling and developmental signaling pathways, with *NOTCH1* among the leading-edge genes, and downregulated antigen presentation pathways and mitochondrial genes. At the gene level, some changes were shared by both donors, including lower *TIMP1*. Because each sex was represented by one donor, these differences cannot be attributed to sex with confidence. The findings are hypothesis-generating and identify candidate pathways for validation in larger, sex-balanced cohorts.

## 1. Introduction

Osteoarthritis (OA) is a debilitating joint disease disproportionately affecting women, especially in postmenopausal years. This disparity suggests a potential role of sex hormones in the pathogenesis and progression of the disease, particularly estrogen [1]. Research indicates that estrogen influences tissue repair and homeostasis, which are critical in joint health. Notably, estrogen’s interaction with matrix metalloproteinases (MMPs), the principal enzymes driving extracellular matrix (ECM) and collagen breakdown, suggests a complex role in maintaining cartilage integrity [2,3].

Estrogen’s protective effects in cartilage are supported by studies demonstrating its ability to suppress MMP expression, which is crucial in preventing ECM degradation, a hallmark of osteoarthritis. For instance, 17β-estradiol (E2) has been shown to reduce the expression of MMPs like *MMP1*, *MMP3*, and *MMP13* in articular chondrocytes, highlighting its potential chondroprotective effects [2]. Moreover, the modulation of MMP activity by estrogen may also involve microRNAs, such as miR-140, which further regulates *MMP13*, a critical enzyme in cartilage matrix breakdown [4].

Despite these protective effects, the role of estrogen in OA is not straightforward. Sex differences in knee biomechanics, such as joint alignment and muscle strength, also contribute to the increased incidence of OA in females compared to males [1]. These biomechanical factors, combined with the molecular effects of estrogen on cartilage and subchondral bone, underline the complexity of OA pathogenesis in different sexes.

The influence of estrogen on meniscal cells, which play a crucial role in load transmission and shock absorption in the knee, remains underexplored. Meniscal fibrochondrocytes (MFCs) exhibit plasticity that is crucial for the repair of meniscal injuries [5], and their response to estrogen may help explain why OA risk differs between women and men. Knewtson and colleagues, whose group generated and deposited the dataset reanalyzed here, reported that E2 alters gene expression in human MFCs in ways that differed between a female and a male donor and between dosing schedules [6]. What has not been resolved is the pathway architecture beneath that response, or whether female and male cells restrain matrix catabolism by the same molecular route. This study compares the effects of 17β-estradiol on MFCs from a female and a male donor, considering both the protective and the potentially deleterious consequences of estrogen exposure for joint health. We reanalyzed a publicly available RNA sequencing dataset (GSE199087) covering an age-matched male and female MFC donor pair, and combined three complementary pathway analysis platforms. We found that E2 engaged distinct transcriptional programs in the cells from the two donors, with the female donor’s profile favoring proliferation and lower expression of matrix-degrading enzymes and the male donor’s profile favoring chromatin remodeling and developmental signaling, alongside a smaller set of changes shared by both. Because the comparison rests on a single donor of each sex, these findings are hypothesis-generating rather than confirmatory. They nonetheless point to candidate pathways for sex-stratified studies of meniscal biology and OA [7].

## 2. Results

### 2.1. Differential Gene Expression Volcano Plot Analysis

#### 2.1.1. Gene-Level Response of the Female Donor’s Meniscal Fibrochondrocytes to 17β-Estradiol

**Figure 1:**
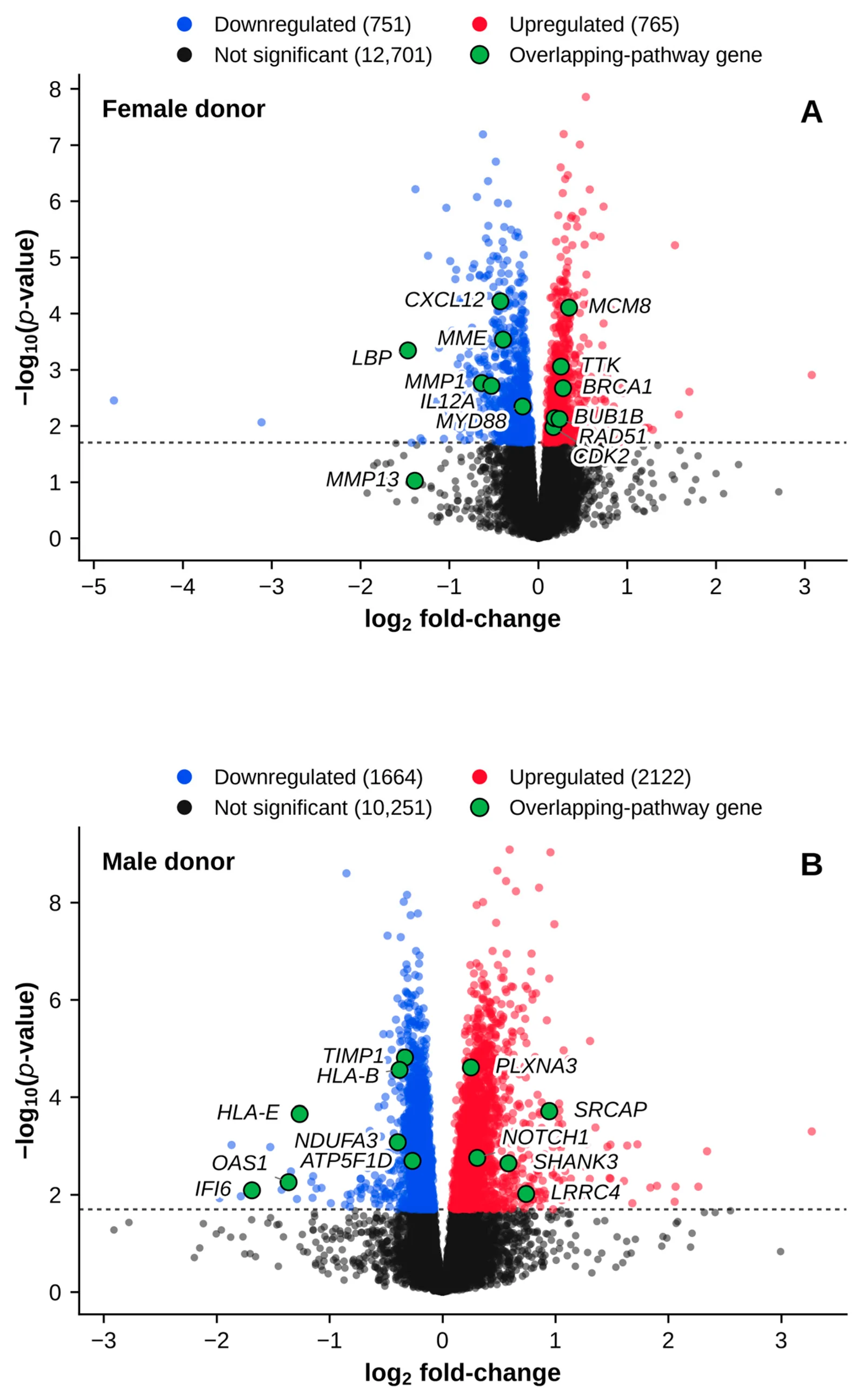
Differential gene expression volcano plots. (**A**) Female donor, E2-treated versus vehicle-treated MFCs. (**B**) Male donor, E2-treated versus vehicle-treated MFCs. The dotted horizontal line marks the significance cut-off of −log_10_(*p*-value) = 1.7 (*p* < 0.02). The *p*-values are nominal and were not corrected for multiple comparisons. Red points are significantly upregulated genes, blue points are significantly downregulated genes, and black points are not significant. The number of genes in each category is given in the legend above each panel. Selected genes from the overlapping pathways (Section 2.3) are labeled and plotted in green. MFC = meniscal fibrochondrocyte; E2 = 17β-estradiol.

Volcano plot analysis (Figure 1A) of the female donor’s MFCs following E2 treatment identified significant differential gene expression, highlighting key upregulated genes involved in cell-cycle regulation and DNA repair (e.g., *MCM8*, *RAD51*, *TTK*, *BRCA1*, *BUB1B*, *CDK2*). In contrast, significantly downregulated genes included factors associated with inflammatory and ECM-degrading processes (e.g., *CXCL12*, *LBP*, *IL12A*, *MYD88*, *MMP1*).

#### 2.1.2. Gene-Level Response of the Male Donor’s Meniscal Fibrochondrocytes to 17β-Estradiol

The volcano plot for the male donor’s MFCs (Figure 1B) showed a distinct gene expression response to E2 treatment. Significantly upregulated genes included those associated with synapse assembly, chromatin remodeling, and developmental signaling (e.g., *PLXNA3*, *SRCAP*, *NOTCH1*, *SHANK3*, *LRRC4*). Conversely, significantly downregulated genes were linked to immune response and mitochondrial function (e.g., *HLA-B*, *HLA-E*, *NDUFA3*, *ATP5F1D*, *OAS1*, *IFI6*).

### 2.2. Overlapping Pathways in Gene Set Enrichment Analysis (GSEA), Enrichr, and iLINCS

#### 2.2.1. Response of the Female Donor’s Meniscal Fibrochondrocytes to 17β-Estradiol

Pathway analyses combining GSEA, Enrichr, and iLINCS supported and extended the differential expression pattern described in Section 2.1.1 (Figure 1A). GSEA results are reported as normalized enrichment scores (NES), and Enrichr and iLINCS results as combined scores (CS). Upregulated pathways included “DNA replication” (GSEA NES = 2.138, Enrichr CS = 70.77, iLINCS CS = 68.19) and “positive regulation of cell cycle process” (GSEA NES = 1.778, Enrichr CS = 304.79, iLINCS CS = 37.68) (Table 1A). Downregulated pathways included “endopeptidase activity” (GSEA NES = −1.586, Enrichr CS = −4.45, iLINCS CS = −7.88) and “fibroblast growth factor receptor signaling pathway” (GSEA NES = −1.755, Enrichr CS = −4.09, iLINCS CS = −5.60), consistent with reports that estrogen limits matrix breakdown in articular cartilage [2,3] (Table 1B).

#### 2.2.2. Response of the Male Donor’s Meniscal Fibrochondrocytes to 17β-Estradiol

Distinct transcriptional responses were observed between the male and female donors following E2 treatment. In the male donor’s MFCs, differentially expressed genes indicated upregulation of factors traditionally associated with synapse assembly, chromatin remodeling, and developmental signaling, such as *PLXNA3*, *SRCAP*, *NOTCH1*, *SHANK3*, and *LRRC4*. Concurrently, immune response genes such as *HLA-B*, *HLA-E*, *OAS1*, and *IFI6* were downregulated (Figure 1B). Integrated analyses highlighted pathways such as “synapse assembly” (GSEA NES = 1.977, Enrichr CS = 10.35, iLINCS CS = 18.49) and “chromatin remodeling” (GSEA NES = 1.766, Enrichr CS = 6.62, iLINCS CS = 23.60) (Table 2A). Downregulated pathways included the major histocompatibility complex (MHC) class I term “antigen processing and presentation of endogenous peptide antigen via MHC class I” (GSEA NES = −2.067, Enrichr CS = −10.12, iLINCS CS = −63.35) and “regulation of peptidase activity” (GSEA NES = −1.547, Enrichr CS = −6.49, iLINCS CS = −14.16), suggesting altered immune and proteolytic responses (Table 2B). The antigen presentation terms reached the three-way overlap only in the male donor’s cells.

#### 2.2.3. Pathway-Level Comparison of the Two Donors

Across all three methods, the female donor’s MFCs predominantly activated proliferative and DNA repair pathways while suppressing inflammatory and ECM-degrading processes (Table 1A,B). The male donor’s MFCs instead upregulated developmental and signaling pathways, with concurrent repression of immune functions and peptidase activity (Table 2A,B). None of the Gene Ontology terms in Table 1 appears in Table 2, although peptidase-related terms were downregulated in both donors, “endopeptidase activity” in the female donor’s cells and “regulation of peptidase activity” in the male donor’s cells.

### 2.3. Leading-Edge Genes in Each Donor

#### 2.3.1. Leading-Edge Gene Analysis in the Female Donor’s MFCs

The leading-edge analysis for the female donor’s MFCs treated with E2 identified the genes that contributed most to the pathway enrichments. Gene-level values are reported as log_2_ fold-change (log_2_FC) with the corresponding nominal *p*-value. For pathways involved in positive regulation of the cell cycle, leading-edge genes included *RGCC* (log_2_FC = 0.525, *p* < 0.001), *CDCA8* (log_2_FC = 0.269, *p* < 0.001), *CDC45* (log_2_FC = 0.302, *p* < 0.001), and *CDC6* (log_2_FC = 0.256, *p* = 0.006). In the DNA replication pathway, *DNA2* (log_2_FC = 0.290, *p* = 0.002) and *CDT1* (log_2_FC = 0.284, *p* = 0.002) were leading-edge members (Table 3A). Conversely, *MYD88* (log_2_FC = −0.179, *p* = 0.004) was a leading-edge member of the downregulated regulation of defense response pathway, and *MME* (log_2_FC = −0.397, *p* < 0.001) was a leading-edge member of the downregulated endopeptidase activity term (Table 3B). Leading-edge membership shows that these genes contributed to the enrichment signal in the female donor’s MFCs. It does not show that they drive pathway activity.

#### 2.3.2. Leading-Edge Gene Analysis in the Male Donor’s MFCs

The leading-edge analysis for the male donor’s MFCs treated with E2 identified the genes contributing to the pathway enrichments. For the “synapse assembly” pathway, leading-edge genes included *PLXNA3* (log_2_FC = 0.250, *p* < 0.001), *SHANK3* (log_2_FC = 0.582, *p* = 0.002), *NOTCH1* (log_2_FC = 0.308, *p* = 0.002), and *LRRC4* (log_2_FC = 0.739, *p* = 0.010). Leading-edge members of the upregulated chromatin remodeling pathway included *ERCC6* (log_2_FC = 0.356, *p* < 0.001), *BICRAL* (log_2_FC = 0.347, *p* < 0.001), *BICRA* (log_2_FC = 0.277, *p* = 0.032), and *SUPT16H* (log_2_FC = 0.136, *p* = 0.020) (Table 4A). Conversely, the downregulated MHC class I antigen presentation pathways included *HLA-E* (log_2_FC = −1.266, *p* < 0.001), *HLA-A* (log_2_FC = −0.279, *p* = 0.016), and *HLA-B* (log_2_FC = −0.382, *p* < 0.001). The “regulation of peptidase activity” pathway included downregulated genes such as the tissue inhibitor of metalloproteinases *TIMP1* (log_2_FC = −0.333, *p* < 0.001), *SPINT2* (log_2_FC = −0.412, *p* = 0.002), *SPCS1* (log_2_FC = −0.182, *p* = 0.001), *SPCS2* (log_2_FC = −0.203, *p* < 0.001), *SEC11A* (log_2_FC = −0.151, *p* = 0.002), *SEC11C* (log_2_FC = −0.268, *p* = 0.021), and *PMPCB* (log_2_FC = −0.099, *p* = 0.014) (Table 4B). *BICRA*, *SUPT16H*, and *SEC11C* did not reach the gene-level cut-off, which is possible for leading-edge genes, as noted under Table 4. These leading-edge genes contributed most to the enrichment signal in the male donor’s MFCs.

### 2.4. Gene-Level Comparison of the Two Donors

**Figure 2:**
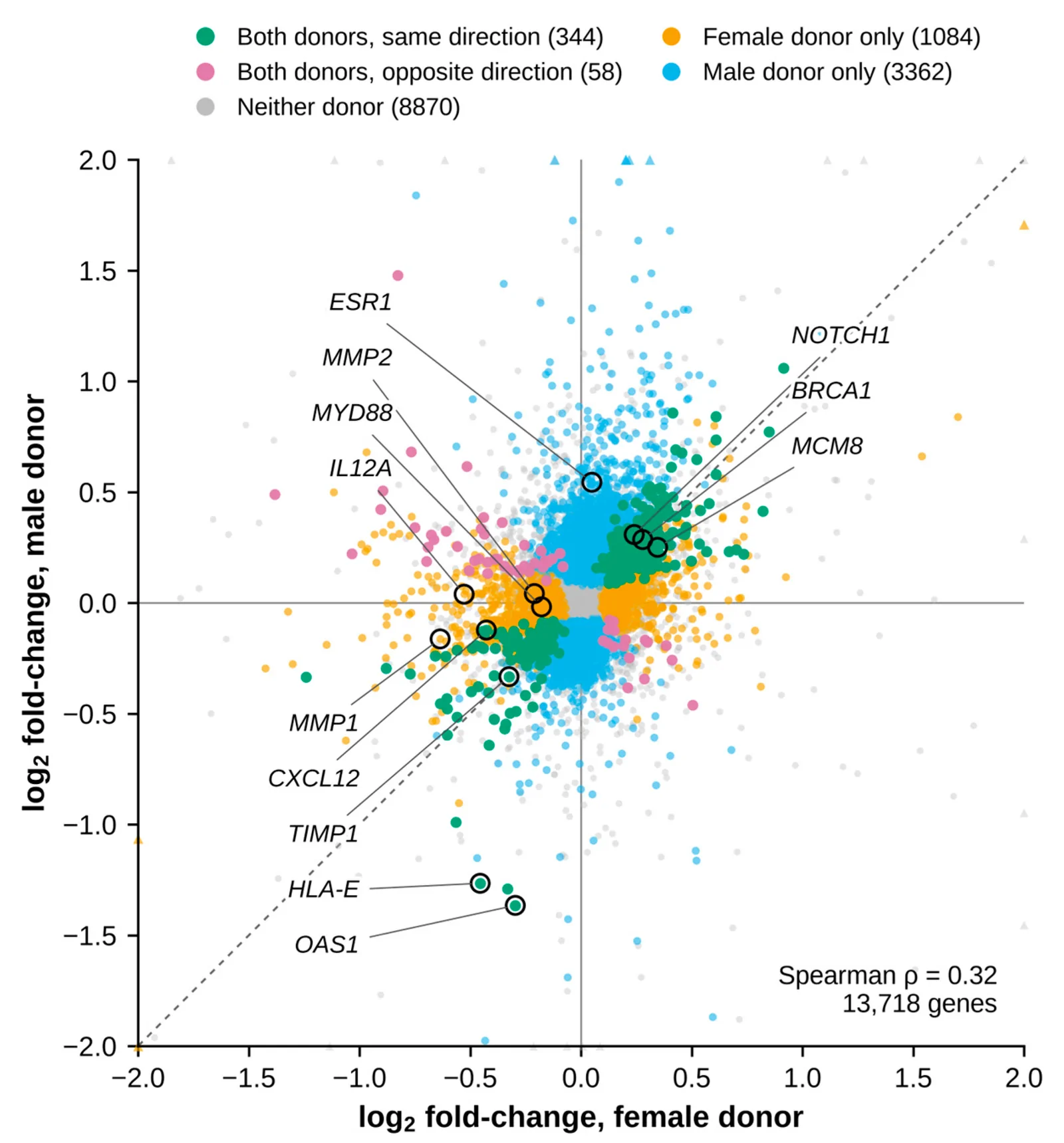
Comparison of the E2 response in the two donors. Each point is one of the 13,718 genes that passed the expression filter in both donors, plotted by its log_2_ fold-change (E2-treated versus vehicle-treated MFCs) in the female donor’s cells (*x*-axis) and the male donor’s cells (*y*-axis). Colors show whether a gene passed the significance cut-off (*p* < 0.02) in both donors in the same direction (green), in both donors in opposite directions (purple), in the female donor only (orange), in the male donor only (blue), or in neither donor (gray). The number of genes in each group is given in the legend above the plot. The dashed line marks equal change in both donors. Selected genes discussed in the text are circled and labeled. The axes are limited to ±2, and the 24 genes beyond this range are drawn as triangles at the edge. MFC = meniscal fibrochondrocyte; E2 = 17β-estradiol.

Of the genes that passed the expression filter, 13,718 were detected in cells from both donors (Figure 2). Of these, 402 passed the significance cut-off in both donors (Table S1). This number is close to the 408 expected by chance from the sizes of the two lists, but 344 of these genes (85.6%) changed in the same direction (223 upregulated and 121 downregulated), well above the 50% expected by chance (binomial test, *p* < 0.001). Across all 13,718 genes, the log_2_FC values of the two donors were positively correlated (Spearman ρ = 0.32, *p* < 0.001). Ten of the 344 genes that changed in the same direction are also leading-edge genes in Table 3 and Table 4. The DNA replication and repair genes *BRCA1*, *CDT1*, *DNA2*, and *MCM8*, which are leading-edge genes of the female donor’s upregulated pathways, also rose in the male donor’s cells. *HLA-A*, *HLA-E*, *OAS1*, *SERPING1*, and *TIMP1*, which are leading-edge genes of the male donor’s downregulated pathways, also fell in the female donor’s cells, and *TIMP1* fell by a similar amount in both donors (log_2_FC = −0.326, *p* = 0.007 in the female donor’s cells and log_2_FC = −0.333, *p* < 0.001 in the male donor’s cells). *CXCL12*, a leading-edge gene of the female donor’s downregulated pathways, also fell in the male donor’s cells. In contrast, *MMP1*, *MMP2*, *IL12A*, *MYD88*, and *MME* changed significantly only in the female donor’s cells, and *HLA-B*, *NOTCH1*, *SRCAP*, *SHANK3*, *PLXNA3*, and *LRRC4* changed significantly only in the male donor’s cells.

Because *MMP1* was prominent in the female response, we also examined the other detected MMPs, the TIMPs, the ADAMTS family, which includes the aggrecanases *ADAMTS4* and *ADAMTS5*, and the main collagen and matrix genes (Table S2). In the female donor’s cells, *MMP1* (log_2_FC = −0.636, *p* = 0.002) and *MMP2* (log_2_FC = −0.211, *p* = 0.002) fell, and *MMP13* showed a large downward trend that did not reach the cut-off (log_2_FC = −1.389, *p* = 0.095). No other MMP reached the cut-off in the female donor’s cells. In the male donor’s cells, *MMP1* and *MMP2* did not change, and the only MMP to reach the cut-off was *MMP23B* (log_2_FC = −1.347, *p* = 0.005), which was below the expression filter in the female donor’s cells. *ADAMTS4* and *ADAMTS5* did not change in either donor, although several other ADAMTS genes did. Within the TIMP family, only *TIMP1* changed, and it fell in both donors. Several other matrix-related genes also changed in both donors, including *CTSK*, *HTRA1*, *PLAU*, and *HAPLN1*, which fell, and *ELN*, *LOXL2*, and *SERPINE1*, which rose. Beyond these shared changes, the structural matrix response differed between the donors. In the male donor’s cells, many collagen and matrix genes rose, including *COL3A1*, *COL4A1*, *COL5A1*, *COL6A3*, *COL12A1*, *FN1*, *FBN1*, *VCAN*, and *TNC*, while *LUM* fell. In the female donor’s cells, fewer structural genes changed, including *COL1A1*, *LOX*, and *SPARC*, which rose, and *DCN* and *PRG4*, which fell. The proteases *CTSB* and *PLAT* also fell only in the female donor’s cells. Three matrix-related genes, *COL14A1*, *COL24A1*, and *CHI3L1*, fell in the female donor’s cells and rose in the male donor’s cells.

*ESR1* and *ESR2*, which encode estrogen receptors α and β, were examined together with *GPER1*, which encodes the G protein-coupled estrogen receptor. *ESR1* transcripts were detected in cells from both donors. E2 increased *ESR1* expression in the male donor’s cells (log_2_FC = 0.544, *p* < 0.001) but not in the female donor’s cells (log_2_FC = 0.049, *p* = 0.749). *ESR2* passed the expression filter in the female donor’s cells (log_2_FC = 0.110, *p* = 0.613) but not in the male donor’s cells, where it did not reach 1 count per million in any sample. *GPER1* was detected in both donors and did not change (log_2_FC = −0.009, *p* = 0.849 in the female donor’s cells and log_2_FC = −0.066, *p* = 0.275 in the male donor’s cells).

## 3. Discussion

This study compares the transcriptomic responses of human MFCs from a female and a male donor to E2 treatment. By reanalyzing RNA sequencing data from dataset GSE199087 and integrating three independent pathway analysis platforms, GSEA, Enrichr, and iLINCS, we found that E2 activated proliferative and DNA repair pathways in the female donor’s MFCs while suppressing inflammatory and matrix-degrading genes. In the male donor’s MFCs, E2 instead upregulated chromatin remodeling and developmental signaling pathways, with concurrent repression of immune functions. A smaller set of changes, including lower *TIMP1* expression, was shared by both donors. If confirmed in more donors, these differences could help explain the well-documented sexual dimorphism in OA prevalence and severity, where females carry a disproportionate disease burden, particularly after menopause [8,9].

### 3.1. Proliferative and Anti-Catabolic Changes in the Female Donor’s MFCs

In the female donor’s MFCs, E2 treatment produced a coordinated upregulation of genes governing cell-cycle progression and DNA repair, including *RGCC*, *MCM8*, *CDC45*, *BRCA1*, *RAD51*, *BUB1B*, and *CDK2* (Table 3A), and the overlapping upregulated pathways centered on DNA replication and cell-cycle progression (Table 1A), indicating a proliferative and genomic maintenance response. Four of the upregulated leading-edge genes in Table 3A, *BRCA1*, *CDT1*, *DNA2*, and *MCM8*, also rose significantly in the male donor’s cells, although the proliferative pathways reached the three-way overlap only in the female donor’s cells (Section 2.4). E2 also lowered genes associated with inflammation and ECM catabolism, including *MMP1*, *LBP*, *IL12A*, *CXCL12*, *MYD88*, and *MME* (Table 3B), and the downregulated pathways converged on endopeptidase activity, fibroblast growth factor receptor signaling, regulation of defense response, and positive regulation of cell death (Table 1B).

This combination of increased proliferative gene expression and reduced catabolic gene expression is consistent with the chondroprotective actions of estrogen reported in articular cartilage. Prior work demonstrated that 17β-estradiol suppresses *MMP3* and *MMP13* mRNA in articular chondrocytes from female patients cultured in a three-dimensional alginate system, with *MMP1* reduced at higher doses [2]. Similarly, E2 reduces MMP1 secretion in OA chondrocytes, particularly in the absence of high-level cytokine stimulation [3]. The downregulation of *MMP1* and *MMP2* in the female donor’s MFCs, alongside a pronounced but non-significant downward trend in *MMP13* (log_2_FC = −1.389, *p* = 0.095), is consistent with these findings and suggests that estrogen’s anti-catabolic effects may extend from articular cartilage to meniscal fibrocartilage, although this was seen in only one of the two donors. Beyond direct MMP suppression, E2 protects chondrocytes through additional mechanisms, including promotion of autophagy and mitophagy via the SIRT1-mediated AMPK/mTOR signaling pathway [10] and regulation of *MMP13* expression through microRNA-140 [4]. Whether these alternative protective mechanisms contribute to the transcriptomic changes observed in the female donor’s MFCs warrants further investigation.

### 3.2. Distinct Transcriptomic Response in the Male Donor’s MFCs

The male donor’s MFCs displayed a markedly different transcriptomic response to E2. Upregulated genes were enriched for synapse assembly, chromatin remodeling, and receptor signaling, with prominent leading-edge genes including *SRCAP*, *SHANK3*, *ERCC6*, *NOTCH1*, *BICRAL*, *BICRA*, and *SUPT16H* (Table 4A). These genes and the overlapping upregulated pathways (Table 2A) suggest that the male donor’s MFCs responded mainly through epigenetic and developmental programs rather than the proliferative and repair-oriented response seen in the female donor’s cells.

Upregulation of *NOTCH1* in the male donor’s MFCs is of particular interest given the central role of Notch signaling in cartilage pathobiology. *NOTCH1* is the most abundantly expressed Notch receptor in articular cartilage, and its expression is elevated in OA, where it drives chondrocyte hypertrophy and terminal differentiation [11,12]. In mouse knee joints, tissue-specific inactivation of the Notch transcriptional effector RBPjκ in adult articular cartilage confers resistance to OA development, while Notch intracellular domain overexpression stimulates endochondral ossification through induction of *Hes1* [11]. Another study demonstrated that silencing *NOTCH1* in human OA chondrocytes reduces proliferation, decreases expression of hypertrophy markers *RUNX2* and *MMP13*, and improves viability in 3D culture [12]. In this context, the E2-mediated upregulation of *NOTCH1* in the male donor’s MFCs may represent a potentially deleterious signal that could promote hypertrophic differentiation rather than tissue maintenance. *NOTCH1* rose by a similar amount in the female donor’s cells but did not reach significance there (log_2_FC = 0.240, *p* = 0.135), so this change may not be specific to the male donor.

On the downregulated side, the male donor’s MFCs showed repression of MHC class I antigen presentation genes (*HLA-A*, *HLA-B*, *HLA-E*) and mitochondrial components (*NDUFA3*, *ATP5F1D*), as well as *TIMP1* (log_2_FC = −0.333, *p* < 0.001) (Table 4B). *HLA-A* and *HLA-E* also fell in the female donor’s cells, by smaller amounts, and *TIMP1* fell there to a similar degree (log_2_FC = −0.326, *p* = 0.007), although it was not a leading-edge gene in the female pathways (Section 2.4). Because TIMPs are endogenous inhibitors of MMPs, the difference between the donors in the MMP/TIMP axis lies in the proteinases rather than in *TIMP1*, since *MMP1* and *MMP2* fell only in the female donor’s cells.

These results can be read against the gene-level analysis of the same donor pair, which applied DESeq2 with a false discovery rate criterion rather than the ranked pathway approach used here [6]. Three points of contact deserve comment. First, they found that male cells yielded more differentially expressed genes than female cells, which agrees with the larger number of significant genes recovered in our male comparison (Figure 1). Second, they reported that the differentially expressed TIMPs, *TIMP2* and *TIMP3*, were downregulated across all treatment groups. In our reanalysis, *TIMP2* and *TIMP3* did not pass the cut-off in either donor, whereas *TIMP1* fell in cells from both donors. Both analyses therefore point to lower TIMP expression after E2, although they differ in which TIMP genes changed. Because *MMP1* and *MMP2* fell only in the female donor’s cells, the male donor’s cells would be left with a less favorable ratio of these proteinases to *TIMP1*, and protein-level measurement of both arms of this axis is needed before that interpretation can be relied upon. Third, they reported that the differentially expressed collagens were downregulated, along with matrix genes such as *LOXL2* and *FBN1*. In our reanalysis, *COL14A1* and *COL24A1* also fell in the female donor’s cells, whereas *COL1A1*, *COL5A3*, and *COL15A1* rose there and several collagen genes rose in the male donor’s cells. *LOXL2* rose in both donors and *FBN1* rose in the male donor’s cells (Table S2). These differences may reflect the different statistical criteria of the two analyses and should be resolved by direct measurement.

### 3.3. Shared and Donor-Specific Responses

With one donor of each sex, no single change can be assigned to sex with confidence. We therefore consider the most likely candidates for a reproducible sex-associated response to be changes that meet three conditions. They pass the cut-off in one donor while remaining essentially unchanged in the other (*p* > 0.1, with an absolute log_2_FC less than half that in the first donor), they are leading-edge genes of pathways supported by all three analysis platforms, and they agree with independent evidence from other human cartilage cells. The suppression of *MMP1* in the female donor’s cells meets all three conditions. It was absent in the male donor’s cells (log_2_FC = −0.163, *p* = 0.433), *MMP1* is a leading-edge gene of the downregulated female pathways (Table 3B), and E2 has been reported to lower *MMP1* in human articular chondrocytes [2,3]. The suppression of *IL12A*, *MYD88*, and *MME* in the female donor’s cells, and the induction of *SHANK3* and *PLXNA3* and repression of *HLA-B* in the male donor’s cells, meet the first two conditions but lack independent support in cartilage. Changes seen in both donors, such as the fall in *TIMP1* and in several interferon-response genes and the rise in several DNA replication genes, are better regarded as responses to E2 shared by both donors than as sex effects. *NOTCH1* may also belong to this shared response, since it rose by a similar amount in both donors. Even changes that meet all three conditions remain candidates, because none of the conditions can separate sex from donor. Distinguishing sex effects from donor effects will require cells from several donors of each sex.

### 3.4. Possible Relevance to Sex Differences and Menopause in OA

If they reflect sex rather than donor, the patterns observed here could help explain the pronounced increase in OA incidence that women experience around menopause. A meta-analysis found that males had a significantly reduced risk for prevalent knee OA compared to females (RR 0.63, 95% CI 0.53–0.75), with females tending toward more severe disease, particularly after menopausal age [8]. More recently, a review reported that women account for approximately 60% of all OA cases globally, with the sex disparity widening after age 40 [9]. The decline in circulating estrogen at menopause has been proposed as a contributing factor to this increased susceptibility, supported by evidence that estrogen deficiency accelerates cartilage degradation and promotes inflammatory cytokine expression in joint tissues [13,14].

One hypothesis consistent with our data is that, in the presence of E2, female MFCs maintain a phenotype characterized by cell renewal and inflammatory suppression, and that the withdrawal of E2 at menopause removes this program, potentially exposing meniscal tissue to catabolic and inflammatory processes. Some epidemiological evidence suggests that hormone replacement therapy may offer modest protection against radiographic knee OA [15,16]. A nationwide Korean cross-sectional study, for example, reported a reduced odds ratio (0.70, 95% CI 0.50–0.99) for knee OA in postmenopausal women receiving menopausal hormone therapy [16], although results across studies remain inconsistent. The dataset contains no premenopausal or postmenopausal comparison, so this hypothesis remains untested.

### 3.5. Estrogen Receptor Expression in Meniscal Tissue

The differential response to E2 between the donors may be partially explained by differences in estrogen receptor expression. In a mouse model, estrogen receptor (ER)-α and ER-β were differentially expressed across fibrocartilaginous tissues, with relatively weak expression of both receptors in knee meniscus cells compared to temporomandibular joint and pubic symphysis cells [17]. Although this study was conducted in mice and its applicability to human meniscal cells remains to be established, it suggests that receptor levels, and therefore responses to E2, may differ between joint tissues. In our data, *ESR1* transcripts were detected in cells from both donors, and E2 increased *ESR1* expression only in the male donor’s cells (Section 2.4). *ESR2* passed the expression filter in the female donor’s cells but not in the male donor’s cells, and *GPER1* was detected in both without change. E2-induced *ESR1* expression could amplify receptor signaling and may contribute to the larger number of genes that changed in the male donor’s cells. Classic estrogen-responsive genes were not strongly induced in either donor. *PGR* did not reach the cut-off in either donor, *GREB1* was below the expression filter in the female donor’s cells and unchanged in the male donor’s cells, and *TFF1* was below the expression filter in both donors, so part of the response may occur through routes other than classical receptor-driven transcription at this concentration. Transcript levels do not establish receptor protein levels or activity, and with one donor of each sex we cannot tell whether these receptor differences reflect sex or donor. Whether receptor density or subtype distribution differs by sex in human meniscal cells therefore remains an important question for future work.

### 3.6. Potential Relevance for OA Research

These findings, if confirmed, have several implications for OA research. First, they support treating sex as a biological variable in studies of estrogen and meniscal biology, since the female and male donors’ cells engaged different programs in response to E2, and OA in human knees has previously been shown to exhibit sex-specific molecular characteristics [7]. Second, the increase in proliferative gene expression and the reduction in catabolic gene expression in the female donor’s cells provide a rationale for testing whether estrogen supports meniscal health in postmenopausal women, though any such approach must be weighed against the broader risk–benefit profile of hormone therapy. Third, the genes identified in each donor are candidates for experimental testing rather than treatment targets. For example, the downregulation of *MMP1* and *MYD88* in the female donor’s cells suggests that anti-catabolic approaches could complement the potentially protective effects observed in the present transcriptomic analysis, while the rise in *NOTCH1*, significant only in the male donor’s cells, points to the Notch pathway, which has been studied in preclinical OA models [11] and reviewed in the context of articular cartilage [18]. Testing a small number of such candidates in cells from more donors would be more informative than pursuing many targets at once.

### 3.7. Limitations

Several limitations warrant consideration. Most importantly, our analysis was based on MFCs derived from one male and one female donor (both aged 47 years), which substantially limits the generalizability of our findings. With one donor of each sex, differences between the two responses could reflect each donor’s genetic background, epigenetic history, or joint health rather than sex. The two donors also differed in reported race, as noted by the investigators who generated the dataset [6]. Donor ancestry therefore cannot be separated from sex as a possible source of the differences reported here, and this categorization is raised solely because it is a potential confounder of the sex comparison. The source study also did not report the donors’ joint condition, medical history, body mass index, nutrition, physical activity, or metabolic status. These factors shape joint tissue in vivo, and a single factor such as high circulating cholesterol could influence part of what we report, since cholesterol metabolism has been shown to regulate cartilage degradation in OA [19]. Second, we analyzed only the pulsed high-dose condition against vehicle control. The low-dose and continuous-dosing groups available in the source dataset were not examined, so dose–response relationships and the effect of dosing mode remain to be characterized. Third, although the pulsed schedule (1 h E2 followed by 23 h E2-free, repeated over 72 h) was designed to mimic cyclical estrogen exposure, the concentration used (240 µM) exceeds physiological circulating estradiol levels, a choice the original investigators made deliberately in order to elicit a measurable transcriptional response [6]. At this concentration, effects that do not depend on estrogen receptors cannot be excluded. Genes that mark heat shock, the unfolded protein response, and oxidative stress (*HSPA1A*, *HSPA1B*, *HSPA5*, *DDIT3*, *XBP1*, *HMOX1*, and *ATF3*) did not pass the cut-off in either donor where detected, which argues against a strong stress response, but the responses described here may not translate directly to physiological estrogen signaling. Fourth, the in vitro monolayer culture contained MFCs alone and does not recapitulate the complex in vivo environment of the knee joint, where MFCs experience mechanical loading, exchange signals with synovial cells, chondrocytes, immune cells, and adipose tissue, and are embedded within an organized extracellular matrix. Our data therefore do not capture the crosstalk between MFCs and the cells that surround them. Fifth, our analysis is based on transcriptomic changes alone, and none of the gene-level results were confirmed by quantitative PCR or at the protein level. Independent confirmation of the key transcripts, followed by protein-level measurement, would be needed to establish functional significance. Sixth, gene-level significance was defined by a nominal *p*-value threshold without correction for multiple comparisons, so individual gene-level results should be read as candidates for validation rather than as established effects. Finally, as a secondary analysis of a publicly available dataset (GSE199087), our study is constrained by the experimental parameters of the original investigators.

### 3.8. Future Directions

Future studies should investigate E2 effects on MFCs from a larger, more diverse cohort of donors to assess reproducibility and capture individual variation, ideally recording each donor’s nutrition, physical activity, and metabolic status. The two adolescent female donors in the same dataset were studied in a separate experiment, so age effects within females would be best examined in new cultures processed together. Quantitative PCR and protein-level validation of key findings, particularly *MMP1*, *MMP2*, *MMP13*, *MYD88*, *NOTCH1*, and *TIMP1*, would strengthen the transcriptomic observations. The use of more physiologically relevant models, such as 3D culture systems, co-culture with synovial cells, or ex vivo meniscal explants, would better approximate the in vivo meniscal environment, and the plasticity of MFCs in such systems has been previously documented [5]. Measuring estrogen receptor protein levels (ER-α, ER-β, and GPER) in human meniscal tissue and relating them to the responses reported here would help clarify the signaling mechanisms involved. Furthermore, examining the effects of other sex hormones, including progesterone and testosterone, on MFC gene expression would provide a more complete picture of hormonal influences on meniscal biology. Longitudinal studies correlating serum estrogen levels, meniscal integrity, and OA progression in human subjects would help validate these in vitro observations clinically.

## 4. Materials and Methods

### 4.1. Dataset

We reanalyzed RNA sequencing data from the dataset GSE199087, available on the Gene Expression Omnibus (GEO) database [20]. The full series characterizes human meniscal fibrochondrocytes (MFCs) from one male (47-year-old) and three female (16-, 17-, and 47-year-old) donors treated with 17β-estradiol (E2) across five treatment groups: vehicle control, pulsed low-dose, pulsed high-dose, continuous low-dose, and continuous high-dose. For the present sex comparison we analyzed the age-matched donor pair from Experiment 1 of that series, comprising MFCs from the knee of a 47-year-old male donor and a 47-year-old female donor, and within that pair we compared the pulsed high-dose condition against the vehicle control. The 16- and 17-year-old female donors were studied in the second experiment of the series, which used a different RNA purification kit, no DNase treatment, and different software to filter the sequencing reads [20]. These donors also have no age-matched male counterpart, so including them would have confounded sex with age and with differences in laboratory methods. Each group was run in triplicate. According to the source study, cells were harvested from the medial meniscus of cadaveric donors obtained through the National Disease Research Interchange, expanded to passage 1, and cultured for 3 days in phenol red-free DMEM with 15% charcoal/dextran-treated fetal bovine serum to remove endogenous hormones before being passaged and seeded at 10,000 cells/cm^2^ in 24-well plates for treatment [6]. Cells were treated with E2 (E2758, Sigma-Aldrich, St. Louis, MO, USA) at the high pulsed dose recorded in the source dataset (240 µM) in phenol red-free DMEM supplemented with 15% charcoal/dextran-treated fetal bovine serum and 1% penicillin/streptomycin. Pulsed dosing was used to resemble the normal estrogen cycle: cells were exposed to E2 for 1 h, after which the E2-containing medium was replaced with E2-free medium for 23 h, and this 24 h cycle was repeated over a total treatment period of 72 h. Total RNA was collected in TRIzol (Life Technologies, Carlsbad, CA, USA) and purified using a PureLink RNA Mini Kit (12183025, Life Technologies), with genomic DNA removed using a DNA-free DNA Removal Kit (AM1906, Life Technologies). Sequencing libraries were prepared with the NEBNext Ultra II Directional RNA Library Prep Kit for Illumina (E7760, New England Biolabs, Ipswich, MA, USA) and sequenced on a NextSeq 550 platform (Illumina, San Diego, CA, USA), with samples run across three flow cells.

### 4.2. Data Processing and Differential Expression Analysis

Transcript-level abundance estimates generated with Kallisto (version 0.46.1) by the investigators who produced the dataset were obtained from GEO [20]. These estimates were summarized to gene level in R using the tximport package, with a transcript-to-gene map derived from the Ensembl 101 annotation of GRCh38 (EnsDb.Hsapiens.v101, retrieved through AnnotationHub) and transcript version suffixes ignored during matching. Gene-level counts were normalized by the trimmed mean of M-values method in edgeR, and genes were retained if their counts per million reached at least 1 in at least one sample. Filtering and model fitting were performed separately for each donor. Retained counts were transformed with voom, and differential expression between the E2-treated and vehicle-treated groups was assessed by fitting a linear model with a group-means design followed by empirical Bayes moderation, using the limma package. We followed published guidance on differential expression and pathway enrichment analysis of public transcriptomic datasets [21,22,23,24].

### 4.3. Volcano Plot

A volcano plot (Figure 1) was generated in Python (version 3.14) using Matplotlib (version 3.11.2) to visualize significantly differentially expressed genes (DEGs). The plot represents differential expression as log_2_ fold-change (log_2_FC) on the *x*-axis against the significance (−log_10_(*p*-value)) on the *y*-axis. Genes above the dotted horizontal threshold (−log_10_(*p*-value) > 1.7, corresponding to *p* < 0.02) were considered statistically significant. Comparable reanalyses of GEO datasets have applied differential expression cut-offs of similar magnitude, typically ranking genes directly by fold-change [25] or pairing a *p*-value threshold of 0.05 with a fold-change filter [26,27]. Gene-level *p*-values reported in this study are nominal; no correction for multiple comparisons was applied at the gene level, and gene-level results are therefore interpreted as hypothesis-generating. Pathway-level significance was assessed on adjusted *p*-values, as described in Section 4.4. Genes were categorized based on log_2_ fold-change, with those significantly upregulated or downregulated highlighted distinctly. Selected genes involved in overlapping pathways identified by pathway analyses were labeled directly on the volcano plots.

### 4.4. Overlapping Pathway Analysis

Pathway analysis was performed in R (version 4.4.0) with Bioconductor release 3.19, using a reproducible reporting pipeline built on BioPathNet (version 0.2.0) that applies three complementary approaches to the same differential expression result. Gene symbols were first reconciled against current HGNC nomenclature with HGNChelper (version 0.8.14), and a fixed random seed (123) was set. First, gene set enrichment analysis was performed on the full ranked gene list using the fgsea implementation of GSEA (version 1.30.0) [21], which returns a normalized enrichment score (NES) and a Benjamini–Hochberg adjusted *p*-value for each Gene Ontology term; terms were retained at an adjusted *p*-value of 0.05 or below. Second, Enrichr was queried through the enrichR package (version 3.2) [22] against the GO_Biological_Process_2023, GO_Molecular_Function_2023, and GO_Cellular_Component_2023 libraries, using the most strongly upregulated and most strongly downregulated genes as selected by log_2_ fold-change, and enriched terms are reported as the Enrichr combined score (CS). Third, the differential expression signature was submitted to iLINCS [23] through the drugfindR package (version 0.99.636) to identify LINCS L1000 chemical perturbagen signatures positively or negatively correlated with the meniscal response to E2. The gene targets of those perturbagens were then themselves tested for Gene Ontology enrichment with Enrichr, and the combined scores from that step are what appear in the iLINCS column of Table 1 and Table 2. GSEA normalized enrichment scores are negative for terms enriched among downregulated genes. Enrichr combined scores are non-negative as returned by the tool; in Table 1 and Table 2, combined scores for terms drawn from the downregulated gene sets are shown with a negative sign so that the direction of change is preserved. Gene Ontology terms were then compared across the three approaches following preparation with PAVER (version 0.0.0.9000), and only terms identified by all three in the same direction were retained. These overlapping terms were ranked by GSEA NES, and the ten most strongly upregulated and ten most strongly downregulated are reported; fewer than ten are shown where fewer terms met the three-way criterion.

### 4.5. Leading-Edge Gene Analysis

GSEA also returns, for each enriched gene set, the leading-edge subset of genes that contributes most to the enrichment score. Gene sets were defined using Gene Ontology annotations [28]. To identify the genes that contributed most consistently to the observed pathway changes, we counted the number of significantly enriched gene sets in which each gene appeared as a leading-edge member, separately for positively and negatively enriched sets. This count is reported as N in Table 3 and Table 4, alongside each gene’s log_2_ fold-change and nominal *p*-value, and reflects how consistently a gene contributes across enriched sets rather than its position in any single one. Genes recurring across many enriched sets were regarded as the most consistent contributors to the enrichment signal and as candidates for experimental validation. Leading-edge membership identifies genes that contribute to an enrichment score. It does not by itself show that a gene drives pathway activity.

### 4.6. Comparison of the Two Donors

Genes that passed the expression filter in both donors were compared directly. A gene was considered changed in both donors when it passed the significance cut-off (−log_10_(*p*-value) > 1.7) in each, and agreement in the direction of change among these genes was tested against the 50% expected by chance with a two-sided binomial test. The number of genes expected to pass the cut-off in both donors by chance was calculated from the number passing it in each donor. The overall similarity of the two responses was summarized by the Spearman correlation of log_2_FC values across all shared genes, which uses ranks and so is not driven by the few genes with very large fold-changes. The genes that passed the cut-off in both donors are listed in Table S1. For the matrix-gene analysis (Table S2), all detected members of the MMP, TIMP, ADAMTS, and collagen families were examined together with selected other matrix-related genes. In each donor’s results, we also examined the estrogen receptor genes *ESR1*, *ESR2*, and *GPER1*, the classic estrogen-responsive genes *PGR*, *GREB1*, and *TFF1*, and markers of heat shock, the unfolded protein response, and oxidative stress (*HSPA1A*, *HSPA1B*, *HSPA5*, *DDIT3*, *XBP1*, *HMOX1*, and *ATF3*). These analyses and Figure 2 were produced in Python (version 3.14) with pandas (version 3.0.6), SciPy (version 1.18.1), and Matplotlib (version 3.11.2).

### 4.7. Figure Preparation and Graphical Abstract

The graphical abstract was created by the authors using Inkscape (version 1.4.3, build 0d15f750, 25 December 2025; Inkscape Project, open-source vector graphics software, available at https://inkscape.org). It is original, previously unpublished artwork, contains no copyrighted or trademarked material, and does not reproduce any figure presented in this manuscript. Figure 1 and Figure 2 were generated directly from the differential expression results in Python as described in Section 4.3 and Section 4.6. No image filters, cropping, or other enhancement techniques were applied to any figure in this manuscript.

## 5. Conclusions

Our reanalysis shows that 17β-estradiol elicited distinct transcriptomic responses in human meniscal fibrochondrocytes from a female and a male donor, together with a smaller shared component that included lower *TIMP1* expression. The female donor’s cells activated proliferative, DNA repair, and cell-cycle pathways while suppressing inflammatory and ECM-degrading genes such as *MMP1* and *MMP2*, a program that would be expected to diminish as estrogen declines at menopause, although this study did not test that inference directly. The male donor’s cells instead engaged chromatin remodeling and developmental signaling pathways, with *NOTCH1* among the leading-edge genes, and repressed antigen presentation pathways. Because each sex was represented by one donor, these differences cannot yet be attributed to sex. They suggest that the anti-catabolic effects of estrogen reported in articular cartilage may extend to meniscal cells, and they identify candidate pathways to test in larger, sex-balanced cohorts.

## Figures and Tables

**Table 1:** Overlapping pathways in GSEA, Enrichr, and iLINCS in the female donor’s MFCs. (**A**) Upregulated pathways. (**B**) Downregulated pathways.

Gene Ontology Term	GSEA (NES)	Enrichr (CS)	iLINCS (CS)
(A) Upregulated pathways			
DNA-templated DNA replication	2.154	100.99	34.40
DNA replication	2.138	70.77	68.19
DNA strand elongation	1.809	48.88	43.69
DNA strand elongation involved in DNA replication	1.803	48.88	43.69
mitotic spindle	1.800	80.19	45.74
positive regulation of cell cycle process	1.778	304.79	37.68
regulation of cell cycle phase transition	1.774	11.94	34.73
regulation of mitotic nuclear division	1.757	71.25	17.05
spindle	1.725	60.22	63.19
mitotic cell cycle checkpoint signaling	1.685	11.94	73.17
(B) Downregulated pathways			
fibroblast growth factor receptor signaling pathway	−1.755	−4.09	−5.60
regulation of vascular associated smooth muscle cell proliferation	−1.590	−12.07	−6.18
growth factor receptor binding	−1.589	−7.88	−6.51
endopeptidase activity	−1.586	−4.45	−7.88
axon guidance	−1.545	−2.66	−6.56
neuron projection guidance	−1.545	−1.63	−7.33
protein processing	−1.429	−4.95	−6.06
axonogenesis	−1.422	−1.35	−12.46
regulation of defense response	−1.323	−3.08	−8.98
positive regulation of cell death	−1.291	−2.38	−6.38

GSEA = gene set enrichment analysis. GSEA values are normalized enrichment scores (NES); Enrichr and iLINCS values are combined scores (CS), shown with a negative sign where the term was enriched among downregulated genes. MFC = meniscal fibrochondrocyte.

**Table 2:** Overlapping pathways in GSEA, Enrichr, and iLINCS in the male donor’s MFCs. (**A**) Upregulated pathways. (**B**) Downregulated pathways.

Gene Ontology Term	GSEA (NES)	Enrichr (CS)	iLINCS (CS)
(A) Upregulated pathways			
synapse assembly	1.977	10.35	18.49
Fc receptor signaling pathway	1.775	12.70	190.53
chromatin remodeling	1.766	6.62	23.60
Fc-epsilon receptor signaling pathway	1.742	18.08	254.55
protein autophosphorylation	1.613	9.65	2493.75
positive regulation of transcription by RNA polymerase II	1.598	7.92	170.34
phosphatidylinositol-mediated signaling	1.591	9.44	1182.28
inositol lipid-mediated signaling	1.569	24.96	445.02
phosphorylation	1.532	10.81	2304.03
cyclic-nucleotide-mediated signaling	1.511	9.44	147.12
(B) Downregulated pathways			
antigen processing and presentation of endogenous peptide antigen via MHC class I	−2.067	−10.12	−63.35
antigen processing and presentation of peptide antigen via MHC class I	−1.929	−7.88	−27.62
negative regulation of protein modification by small protein conjugation or removal	−1.672	−2.43	−9.10
regulation of mitochondrial outer membrane permeabilization involved in apoptotic signaling pathway	−1.593	−3.51	−27.62
regulation of peptidase activity	−1.547	−6.49	−14.16

GSEA = gene set enrichment analysis. GSEA values are normalized enrichment scores (NES); Enrichr and iLINCS values are combined scores (CS), shown with a negative sign where the term was enriched among downregulated genes. MFC = meniscal fibrochondrocyte.

**Table 3:** Leading-edge genes in GSEA for the female donor’s MFCs. (**A**) Upregulated leading-edge genes. (**B**) Downregulated leading-edge genes.

Gene Symbol	Gene Name	N	log_2_FC	*p*-Value
(A) Upregulated leading-edge genes				
*RGCC*	regulator of cell cycle	21	0.525	<0.001
*MCM8*	minichromosome maintenance 8 homologous recombination repair factor	35	0.346	<0.001
*CDC45*	cell division cycle 45	28	0.302	<0.001
*DNA2*	DNA replication helicase/nuclease 2	43	0.290	0.002
*CDT1*	chromatin licensing and DNA replication factor 1	33	0.284	0.002
*BRCA1*	BRCA1 DNA repair associated	41	0.278	0.002
*CDCA8*	cell division cycle associated 8	40	0.269	<0.001
*CDC6*	cell division cycle 6	36	0.256	0.006
*TTK*	TTK protein kinase	51	0.256	<0.001
*RAD51*	RAD51 recombinase	63	0.238	0.008
*BUB1B*	BUB1 mitotic checkpoint serine/threonine kinase B	44	0.184	0.007
*CDK2*	cyclin dependent kinase 2	37	0.169	0.010
(B) Downregulated leading-edge genes				
*LBP*	lipopolysaccharide binding protein	30	−1.468	<0.001
*MMP13*	matrix metallopeptidase 13	12	−1.389	0.095
*MMP1*	matrix metallopeptidase 1	10	−0.636	0.002
*IL12A*	interleukin 12A	41	−0.528	0.002
*CXCL12*	C-X-C motif chemokine ligand 12	33	−0.428	<0.001
*MME*	membrane metalloendopeptidase	14	−0.397	<0.001
*MYD88*	MYD88 innate immune signal transduction adaptor	25	−0.179	0.004
*MMP11*	matrix metallopeptidase 11	6	−0.154	0.463

GSEA = gene set enrichment analysis. Leading-edge genes are drawn from the ranked GSEA list and may therefore include genes above the *p* < 0.02 significance cut-off. MFC = meniscal fibrochondrocyte; N = number of significantly enriched Gene Ontology gene sets in which the gene appears as a leading-edge member; log_2_FC = log_2_ fold-change.

**Table 4:** Leading-edge genes in GSEA for the male donor’s MFCs. (**A**) Upregulated leading-edge genes. (**B**) Downregulated leading-edge genes.

Gene Symbol	Gene Name	N	log_2_FC	*p*-Value
(A) Upregulated leading-edge genes				
*SRCAP*	Snf2 related CREBBP activator protein	21	0.943	<0.001
*LRRC4*	leucine rich repeat containing 4	10	0.739	0.010
*SHANK3*	SH3 and multiple ankyrin repeat domains 3	47	0.582	0.002
*ERCC6*	ERCC excision repair 6, chromatin remodeling factor	27	0.356	<0.001
*BICRAL*	BICRA like chromatin remodeling complex associated protein	1	0.347	<0.001
*NOTCH1*	notch receptor 1	34	0.308	0.002
*BICRA*	BRD4 interacting chromatin remodeling complex associated protein	1	0.277	0.032
*PLXNA3*	plexin A3	37	0.250	<0.001
*SUPT16H*	SPT16 homolog, facilitates chromatin remodeling subunit	2	0.136	0.020
(B) Downregulated leading-edge genes				
*IFI6*	interferon alpha inducible protein 6	24	−1.689	0.008
*OAS1*	2′-5′-oligoadenylate synthetase 1	63	−1.365	0.005
*HLA-E*	major histocompatibility complex, class I, E	85	−1.266	<0.001
*SPINT2*	serine peptidase inhibitor, Kunitz type 2	2	−0.412	0.002
*NDUFA3*	NADH:ubiquinone oxidoreductase subunit A3	65	−0.396	<0.001
*HLA-B*	major histocompatibility complex, class I, B	50	−0.382	<0.001
*TIMP1*	TIMP metallopeptidase inhibitor 1	13	−0.333	<0.001
*CST3*	cystatin C	12	−0.307	<0.001
*HLA-A*	major histocompatibility complex, class I, A	78	−0.279	0.016
*SEC11C*	SEC11 homolog C, signal peptidase complex subunit	4	−0.268	0.021
*ATP5F1D*	ATP synthase F1 subunit delta	73	−0.268	0.002
*SPCS2*	signal peptidase complex subunit 2	7	−0.203	<0.001
*SPCS1*	signal peptidase complex subunit 1	9	−0.182	0.001
*SERPING1*	serpin family G member 1	7	−0.160	<0.001
*SEC11A*	SEC11 homolog A, signal peptidase complex subunit	3	−0.151	0.002
*PMPCB*	peptidase, mitochondrial processing subunit beta	4	−0.099	0.014

GSEA = gene set enrichment analysis. Leading-edge genes are drawn from the ranked GSEA list and may therefore include genes above the *p* < 0.02 significance cut-off. MFC = meniscal fibrochondrocyte; N = number of significantly enriched Gene Ontology gene sets in which the gene appears as a leading-edge member; log_2_FC = log_2_ fold-change.

## Data Availability

The data presented in this study are available in the Gene Expression Omnibus (GEO) at https://www.ncbi.nlm.nih.gov/geo/ (accessed on 8 August 2026), accession number GSE199087. These data were derived from the following resource available in the public domain: https://www.ncbi.nlm.nih.gov/geo/query/acc.cgi?acc=GSE199087 (accessed on 8 August 2026).

## Supplementary Materials

The following supporting information can be downloaded at: https://www.mdpi.com/article/10.3390/ijms27219881/s1.
